# Multidrug-Resistant Serratia fonticola Causing Diabetic Foot Infection: A Rare Case Highlighting Emerging Antimicrobial Resistance

**DOI:** 10.7759/cureus.105964

**Published:** 2026-03-27

**Authors:** Ashish Jangid, Shalini Singh, Prashant Pandey, Dilip Chaurasiya, Shobhit Agarwal

**Affiliations:** 1 General Medicine, Heritage Institute of Medical Sciences, Varanasi, IND; 2 Microbiology, Heritage Institute of Medical Sciences, Varanasi, IND; 3 Internal Medicine, Heritage Institute of Medical Sciences, Varanasi, IND

**Keywords:** ampc beta-lactamase, carbapenem resistance, diabetic foot infection, multidrug resistance, serratia fonticola

## Abstract

Diabetic foot infections are commonly caused by Gram-positive cocci and Enterobacteriaceae. Isolation of *Serratia fonticola* is very rare. Here, we report a case of multidrug-resistant *Serratia fonticola* isolated from a chronic diabetic foot ulcer in a patient with poorly controlled diabetes. The case highlights the importance of microbiological evaluation in non-healing wounds and raises awareness of unusual resistant pathogens in routine clinical practice. A 56-year-old female patient with poorly controlled diabetes developed a non-healing foot ulcer following minor trauma. Culture revealed multidrug-resistant *Serratia fonticola*. The patient improved with glycemic control, debridement, and culture-directed antibiotics.

## Introduction

Diabetic foot infection is one of the most serious complications of diabetes mellitus and a common reason for hospital admission. It is estimated that approximately 15-25% of patients with diabetes develop a foot ulcer during their lifetime [1], and a significant proportion of these ulcers become infected. These infections are associated with considerable morbidity, prolonged hospital stay, and, in severe cases, an increased risk of limb loss and mortality.

Diabetic foot infections are usually polymicrobial in nature. The most commonly isolated organisms include *Staphylococcus aureus*, *Pseudomonas aeruginosa*, and members of the Enterobacteriaceae family [2]. The microbial profile often varies depending on factors such as the duration of the ulcer, prior antibiotic use, and local epidemiological patterns.

Several factors contribute to the development of diabetic foot infections, including peripheral neuropathy, peripheral arterial disease, poor glycemic control, and repeated minor trauma. These conditions impair local defense mechanisms and create an environment that favors colonization and infection. Delayed diagnosis or inappropriate antimicrobial therapy can further worsen outcomes and increase the risk of complications such as deep tissue infection or amputation.

*Serratia fonticola* was initially identified as an environmental organism isolated from water sources [3]. Although traditionally considered non-pathogenic, it is increasingly recognized as an opportunistic pathogen, particularly in individuals with underlying comorbidities. Infections caused by *Serratia* species have gained attention due to their evolving antimicrobial resistance patterns, including intrinsic resistance characteristics and inducible β-lactamase production [4-6].

However, infections due to *Serratia fonticola* remain rare, with only sporadic cases reported, and its role in diabetic foot infections remains poorly documented. This case highlights the importance of considering unusual multidrug-resistant organisms in non-healing diabetic foot ulcers and emphasizes the role of timely microbiological evaluation in guiding appropriate and effective therapy.

## Case presentation

**Figure 1:**
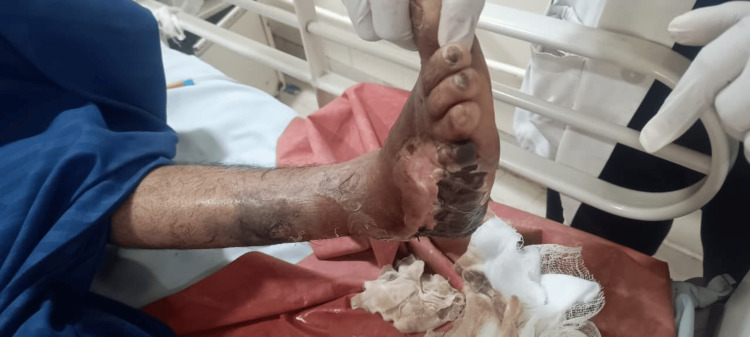
Clinical photograph of the right diabetic foot ulcer.

A 56-year-old female with poorly controlled type 2 diabetes mellitus presented with a non-healing ulcer over the right foot following minor trauma 28 days earlier. She had received treatment at multiple centers before referral. Over the preceding 10 days, the wound developed purulent discharge. Clinical examination revealed a necrotic ulcer with surrounding erythema and seropurulent discharge (Figure 1). The ulcer measured approximately 10 × 6 cm, involving the plantar aspect of the right foot, with a necrotic base and surrounding erythema. The depth of the ulcer was approximately 1-2 cm, with no exposed bone. The patient was febrile (temperature: 101.2°F) at presentation but became afebrile following initiation of treatment and remained hemodynamically stable thereafter. Peripheral pulses were palpable on clinical examination, suggesting adequate vascular perfusion. Sensory examination revealed distal sensory loss with reduced vibration and light touch sensation, consistent with diabetic peripheral neuropathy. Nerve conduction studies of all four limbs were suggestive of sensorimotor axonal polyneuropathy.

**Figure 2:**
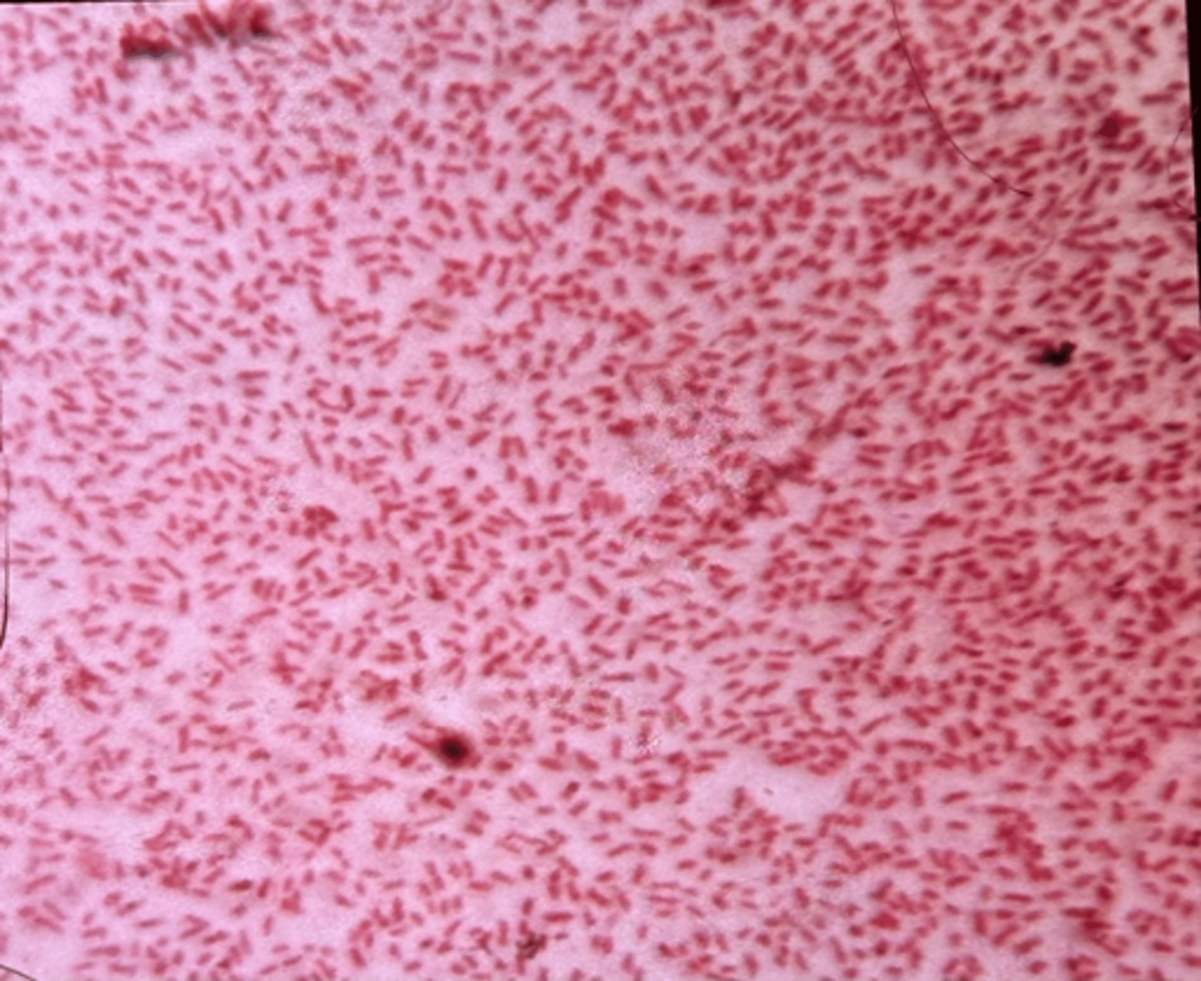
Gram stain showing Gram-negative bacilli.

Pus samples were collected under aseptic precautions. Gram staining demonstrated Gram-negative bacilli (Figure 2).

**Figure 3:**
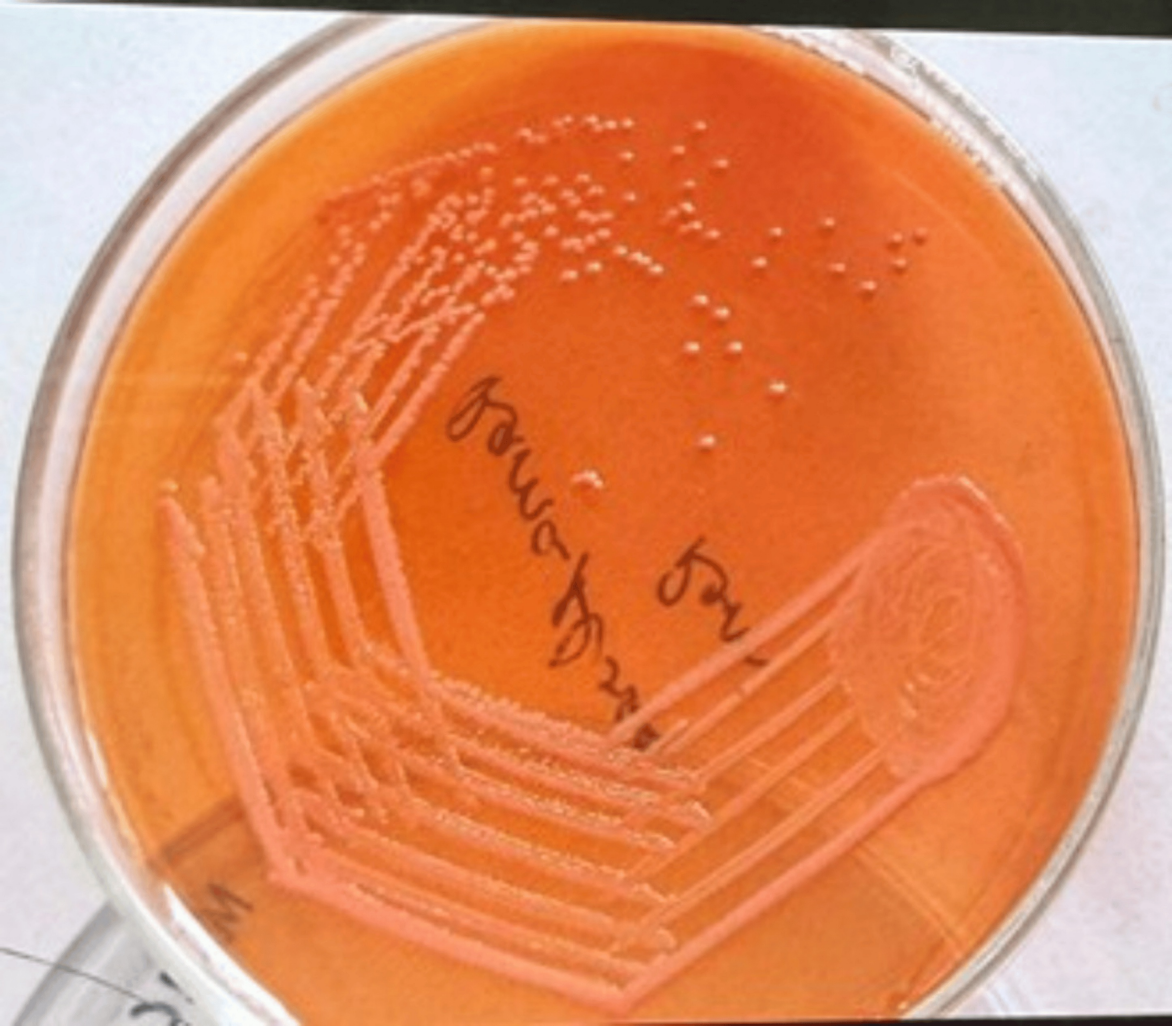
Growth of Serratia fonticola on MacConkey agar showing non-lactose fermenting colonies.

**Figure 4:**
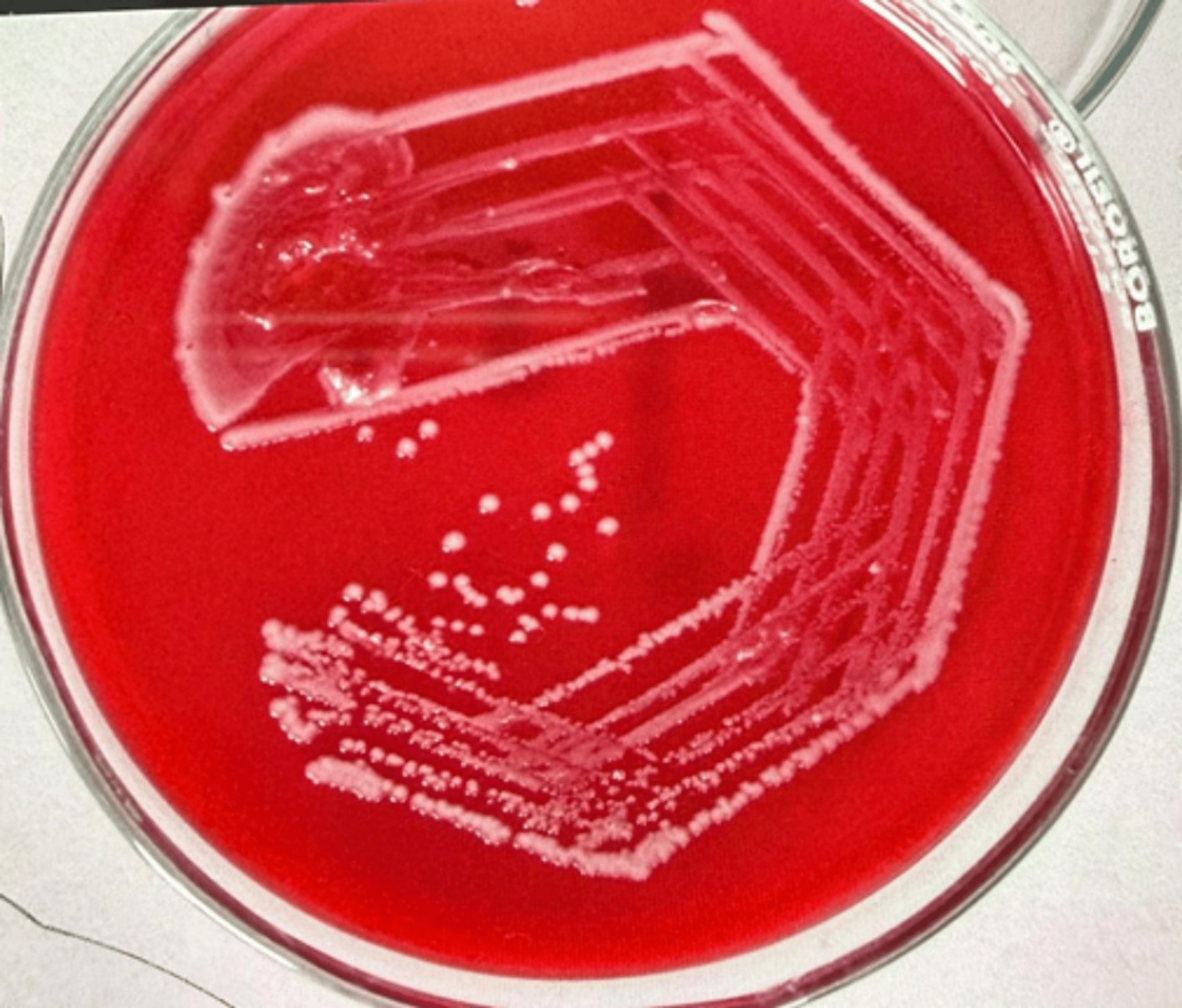
Growth on blood agar showing non-hemolytic colonies.

Culture showed non-lactose fermenting colonies on MacConkey agar (Figure 3) and non-hemolytic colonies on blood agar (Figure 4).

A detailed laboratory evaluation was performed at admission, and the results are summarized in Table 1. Hemoglobin was 10.2 g/dL, and the total leukocyte count was elevated at 15,600/mm³ with neutrophilic predominance. Platelet count and red cell indices were within normal limits. Renal and liver function tests were within reference ranges. Despite leukocytosis, blood cultures obtained at admission remained sterile after 48 hours of incubation.

**Table 1:** Detailed laboratory parameters at admission. AST = aspartate aminotransferase; SGOT = serum glutamic oxaloacetic transaminase; ALT = alanine aminotransferase; SGPT = serum glutamate pyruvate transaminase

Parameter	Patient value	Normal reference range
Complete blood count		
Hemoglobin	10.2 g/dL	12–15 g/dL
Total leukocyte count	15,600/mm³	4,000–11,000/mm³
Neutrophils	78%	40–75%
Lymphocytes	18%	20–40%
Monocytes	3%	2–8%
Eosinophils	1%	1–6%
Basophils	0%	0–1%
Platelet count	2.6 lakh/mm³	1.5–4.5 lakh/mm³
Packed cell volume	34%	36–46%
Mean corpuscular volume	86 fL	80–100 fL
Mean corpuscular hemoglobin	28 pg	27–32 pg
Mean corpuscular hemoglobin concentration	33 g/dL	32–36 g/dL
Renal function tests		
Blood urea	28 mg/dL	15–40 mg/dL
Serum creatinine	0.9 mg/dL	0.6–1.2 mg/dL
Serum sodium	138 mEq/L	135–145 mEq/L
Serum potassium	4.3 mEq/L	3.5–5.0 mEq/L
Serum chloride	102 mEq/L	98–106 mEq/L
Liver function tests		
Total bilirubin	0.8 mg/dL	0.2–1.2 mg/dL
Direct bilirubin	0.2 mg/dL	0–0.3 mg/dL
AST (SGOT)	24 U/L	10–40 U/L
ALT (SGPT)	28 U/L	7–56 U/L
Alkaline phosphatase	92 U/L	44–147 U/L
Total protein	7.1 g/dL	6.0–8.3 g/dL
Serum albumin	3.9 g/dL	3.5–5.0 g/dL
Globulin	3.2 g/dL	2.0–3.5 g/dL
HbA1c	10.2%	<5.7%

The organism was identified as *Serratia fonticola* using a VITEK 2 compact automated identification system. Antimicrobial susceptibility testing was performed by using minimum inhibitory concentration-based methods, and results were interpreted according to Clinical and Laboratory Standards Institute guidelines. The antimicrobial susceptibility testing results are summarized in Table 2.

**Table 2:** Antimicrobial susceptibility pattern. MIC = minimum inhibitory concentration

Antibiotic	MIC	Interpretation
Piperacillin-tazobactam	64	Resistant
Ceftazidime	8	Intermediate
Cefoperazone-sulbactam	<8	Sensitive
Cefepime	≥32	Resistant
Aztreonam	≥64	Resistant
Imipenem	≥16	Resistant
Meropenem	≥16	Resistant
Amikacin	32	Intermediate
Gentamicin	>16	Resistant
Ciprofloxacin	>4	Resistant
Levofloxacin	>8	Resistant
Minocycline	16	Resistant
Tigecycline	>8	Resistant
Colistin	>16	Resistant

The patient underwent surgical debridement of the necrotic tissue. Based on susceptibility results, she was treated with intravenous cefoperazone sulbactam for 14 days along with strict glycemic control using insulin therapy. The wound gradually improved with a reduction in discharge and healthy granulation tissue formation. She was discharged in stable condition and remained clinically stable at the two-week follow-up with progressive wound healing.

## Discussion

*Serratia fonticola* is a Gram-negative bacillus that was first described as an environmental organism isolated from water sources [3]. Although it was historically considered non-pathogenic, it is now increasingly recognized as a potential opportunistic pathogen, particularly in vulnerable individuals such as those with chronic illnesses or impaired immunity. Its isolation in a diabetic foot infection is uncommon and clinically significant, especially in the setting of rising antimicrobial resistance.

A notable feature of *Serratia fonticola* is its intrinsic and acquired resistance potential. The organism carries an inducible chromosomal AmpC beta-lactamase, which can reduce susceptibility to extended-spectrum cephalosporins and aztreonam [4]. Exposure to beta-lactam antibiotics may enhance enzyme expression, thereby compromising therapeutic effectiveness. Furthermore, environmental organisms within the *Serratia* genus have the capacity to acquire additional resistance determinants through horizontal gene transfer, contributing to their evolving resistance profiles [5,6]. The organism showed a multidrug-resistant profile, with resistance to multiple antibiotic classes, including beta-lactams, carbapenems, fluoroquinolones, and polymyxins.

Resistance to carbapenems in *Serratia* species is typically multifactorial and may involve enzymatic degradation, altered membrane permeability, and efflux mechanisms [5,6]. The high minimum inhibitory concentrations observed in our isolate likely reflect the combined action of these mechanisms. The resistance to colistin observed in this case may be explained by intrinsic structural properties of the bacterial outer membrane, which reduce susceptibility to polymyxins.

In diabetic foot ulcers, repeated antibiotic exposure, compromised vascular supply, and persistent hyperglycemia create a favorable environment for the selection of resistant organisms. Although *Serratia fonticola* remains a rare cause of diabetic foot infection, this case highlights the importance of obtaining culture confirmation and tailoring antimicrobial therapy based on susceptibility results in chronic or non-healing wounds. In this case, the patient remained clinically stable at the two-week follow-up with progressive wound healing. This shows the importance of routine microbiological evaluation in chronic or non-healing diabetic foot ulcers, as it enables the identification of unusual multidrug-resistant pathogens and facilitates targeted antimicrobial therapy for better outcomes.

Similar multidrug-resistant cases have been reported in previously described *Serratia* infections, particularly in opportunistic settings, supporting the emerging clinical significance of this organism. The observed resistance to beta-lactams and cephalosporins may be attributed to AmpC beta-lactamase production, while carbapenem resistance may involve additional mechanisms such as reduced membrane permeability and efflux activity. Molecular characterization of resistance genes was not performed, which is a limitation of this report. This case also highlights the importance of infection control practices and surveillance for emerging multidrug-resistant organisms in the healthcare setting.

This case highlights the importance of structured antimicrobial stewardship programs, which have been shown to reduce unnecessary antibiotic use while preserving clinical outcomes, supporting data-driven prescribing practices [7].

## Conclusions

*Serratia fonticola* may be a multidrug-resistant opportunistic pathogen in cases of diabetic foot infection. The expression of AmpC beta-lactamase and lower susceptibility to carbapenem underline the complexity of the treatment of this organism. Early diagnosis, treatment based on culture, and judicious use of antimicrobials are critical to achieve the best outcome in patients and reduce the development of additional resistance. *Serratia fonticola* is an uncommon but possible opportunistic pathogen in diabetic foot infections. Microbiological diagnosis and specific antimicrobial treatment during the treatment of atypical and resistant infections are a priority. As a single case report, the findings may have limited generalizability but provide important clinical insights into emerging multidrug-resistant pathogens.

## References

[1] Armstrong DG, Boulton AJ, Bus SA (2017). Diabetic foot ulcers and their recurrence. N Engl J Med.

[2] Lipsky BA, Senneville É, Abbas ZG (2020). Guidelines on the diagnosis and treatment of foot infection in persons with diabetes (IWGDF 2019 update). Diabetes Metab Res Rev.

[3] Gavini F, Ferragut C, Izard D, Trinel PA, Leclerc H, Lefebvre B, Mossel DAA (1979). Serratia fonticola, a new species from water. Int J Syst Evol Microbiol.

[4] Stock I, Burak S, Sherwood KJ, Gruger T, Wiedemann B (2003). Natural antimicrobial susceptibilities of strains of 'unusual' Serratia species: S. ficaria, S. fonticola, S. odorifera, S. plymuthica and S. rubidaea. J Antimicrob Chemother.

[5] Mahlen SD (2011). Serratia infections: from military experiments to current practice. Clin Microbiol Rev.

[6] Samonis G, Vouloumanou EK, Christofaki M (2011). Serratia infections in a general hospital: characteristics and outcomes. Eur J Clin Microbiol Infect Dis.

[7] Sallam M, Snygg J (2023). Improving antimicrobial stewardship program using the lean Six Sigma methodology: a descriptive study from Mediclinic Welcare Hospital in Dubai, the UAE. Healthcare (Basel).

